# Cancer-associated fibroblasts: key determinants of tumor immunity and immunotherapy

**DOI:** 10.1016/j.coi.2020.03.004

**Published:** 2020-05-11

**Authors:** Richard Barrett, Ellen Puré

**Affiliations:** University of Pennsylvania, Philadelphia, PA 19104, United States

## Abstract

Immune-targeted approaches are rapidly changing the therapeutic landscape for cancer. In spite of that, most patients show resistance or acquire resistance to these therapies. Increasing work describing the tumor microenvironment (TME) has highlighted this space as one of the key determinants in tumor immune response and immunotherapeutic success. Frequently overlooked within this space, cancer-associated fibroblasts (CAFs) within the TME have surfaced as an important dictator of the tumor immune response. Herein, we review recent advances in defining the role of CAF-immune cell interactions in solid tumors and prospects for targeting stroma to overcome resistance to immunotherapy.

## Introduction

Cancer immunotherapies have shown marked therapeutic success as of late and generally fall into two broad categories. The first category are designed to enhance endogenous anti-tumor immunity and include vaccines [1], immune agonists like anti-CD40 [2], and inhibitors of immune checkpoints such as anti-CTLA-4 [3] and anti-PD-1/PD-L1 [4]. However, increasing clinical experience indicates that in spite of the remarkable success of these approaches across multiple tumor types, the majority of patients are either resistant or acquire resistance [5]. The second category involves adaptive cell therapies (ACT) such as chimeric antigen receptor T-Cell (CAR-T) therapy [6,7]. Remarkable successes have also been achieved with ACT; however, these have been limited to hematologic tumors, while proof of their utility in solid tumors remains elusive. Therapeutic resistance is also a major barrier in the context of chemotherapy and targeted therapies. It is therefore imperative that the mechanistic basis of resistance be determined to facilitate the rational design of approaches to avoid or overcome therapeutic resistance. Tumor stroma, comprises mesenchyme-derived stromal cells such as cancer-associated fibroblasts (CAFs) and extracellular matrix (ECM), is complicit in tumor initiation, progression and metastasis. Moreover, stroma represents a major barrier to therapeutic efficacy and has recently been identified as a critical mediator of immune suppression in the tumor microenvironment (TME) [8].

Although fibroblasts were historically largely overlooked in the context of cancer, together with ECM, they are now understood to provide biochemical and biomechanical signals critical to malignant cell behavior. CAFs also impact inflammatory and immune cell infiltration and intra-tumoral migration and contribute to the immune suppressive milieu that typically dominates the microenvironment of advanced solid tumors. In this review, we outline recent advances in the understanding of how fibroblasts influence tumor immunity and how this in turn influences the success of immunotherapies. We also discuss current attempts at therapeutically targeting CAFs.

## The ‘Stromagenic switch’

### Fibroblast activation

Fibroblasts represent a heterogeneous population of mesenchyme-derived cells prominent in all connective tissues. In the broadest sense, fibroblasts can be divided into two primary states, quiescent and activated, although they exhibit significant context-dependent phenotypical and functional diversity.

Fibroblasts under homeostatic conditions exist in most tissues in a relatively quiescent state, referring to their low proliferative capacity and metabolic state. Fibroblast activation is an early response to disruptions in homeostasis, characterized by increased proliferative capacity, increased synthetic activity including production of a provisional matrix, and increased metabolic activity, all designed to restore homeostasis [9].

In the TME, cancer cells can drive fibroblast activation. A number of tumor cell secreted factors can activate fibroblasts including TGFβ, PDGF, EGF, CTGF, and FGF [10,11]. Fibroblasts are also responsive to substratum composition and stiffness with matrix stiffening being associated with fibroblast activation [12^•^]. Stiffness within compliant normal tissues typically ranges from ~.05—5 kPa [13], while progressive stiffening of tumor tissue can reach up to ~20 kPa [14] in the most desmoplastic tumors such as pancreatic cancer. Together, these signals can also drive dedifferentiation of other mesenchymal cell types, such as pericytes and adipocytes, to a CAF-like state [15,16].

### Heterogeneity in fibroblasts

The heterogeneity of CAFs is just beginning to be defined on a molecular level and recent work has been vital in untangling confounding results from earlier studies. Because of initial reports establishing correlations between the prevalence of CAFs and poor prognosis [17,18], a simple paradigm emerged that CAFs are pro-tumorigenic. However, early studies targeting myofibroblasts in the context of pancreatic cancer unexpectedly enhanced tumor progression [19,20]. These seemingly paradoxical observations highlighted a need to better understand the functional diversity of CAFs.

Activated fibroblasts within TME have traditionally been identified based on their expression of alpha smooth muscle actin (α-SMA), and generically referred to as myofibroblasts [21]. More recent studies, however, highlight that α-SMA^+^ cells represent only a subset of all stromal cells within the TME and that CAFs are in fact heterogeneous based on cell surface markers, gene expression profiling, and functionality. To date, neither a unifying approach to defining, nor a standardized nomenclature for CAF subpopulations has yet emerged but promises to be complex based on the evidence that the state of fibroblast activation is both context-dependent, plastic and likely fall along a continuum rather than into discrete subsets. Nonetheless, multiple markers including fibroblast activation protein (FAP) [22], podoplanin (PDPN) [23], fibroblast-specific protein 1 (FSP-1) [24], meflin [25], and platelet-derived growth factor receptor (PDGFR) [24] have surfaced to describe CAF populations with key functional differences within the TME.

Independently, two subpopulations referred to as myCAFs and iCAF have been described in pancreatic and recently other cancer types [26^••^]. MyCAFs (like myofibroblasts) are the traditional α-SMA expressing population and inflammatory fibroblasts (iCAFs) are defined by expression of inflammatory cytokines such as IL-6 and CXCL12 [26^••^,^27^]. These subpopulations also segregate spatially within TME of pancreatic cancer, with myCAFs primarily tumor adjacent, and iCAFs more distal from the edge of tumor nests [27]. These subpopulations overlap significantly with many markers described above. Moreover, single cell sequencing analyses of various tumor types indicate that fibroblasts segregate into anywhere from three to seven clusters based on transcriptome [26^••^,^28^,^29^^•^]. Such analyses are proving useful in highlighting the primary characteristics of each subpopulation and may hopefully lead to more exclusive markers for each functional type.

## CAF – immune cell interactions

A major impact of CAFs on the TME is through their immunomodulatory capacity. Fibroblasts are able to direct and coordinate immune cell infiltration either directly—via secreted cytokines and surface proteins— or indirectly—through deposition of various ECM substrates and remodeling of matrix. It should be noted that some tumor-associated macrophages (TAMs) can also contribute significantly to matrix remodeling in the TME [30]. Understanding these interactions is vital considering the recent explosion of immunotherapies, as CAFs not only influence *de novo* immune responses, but also dictate the success of immunotherapies through these mechanisms.

### CAF influence on myeloid cells

Clues that CAFs play a critical role in immunosuppression came from clinical data showing correlations in expression of stromal markers with infiltration of immunosuppressive cell types such as TAMs and myeloid-derived suppressor cells (MDSC) [31]. MDSC correlate not only with poorer overall survival across a variety of cancers, but also with disease resistance to immunotherapy [17,32].

Myeloid cells in TME are known to drive immunosuppression including suppression of cytotoxic T-cell activity [33]. CAFs secrete many signaling molecules known to influence both recruitment and activation state of myeloid cells including: CXCL1, CXCL2, CXCL5, CXCL6/GCP-2, CXCL8, CXCL9, CXCL10, CXCL12/SDF1, CCL2/MCP-1, CCL3, CCL5/Rantes, CCL7, CCL20, CCL26, IL-1β, IL-6, IL-10, VEGF, TGF-β, indoleamine-2,3-dioxygenase (IDO), prostaglandin (PG) E2 (PGE2), tumor necrosis factor (TNF) or nitric oxide (NO) [34,35].

**Figure 1:**
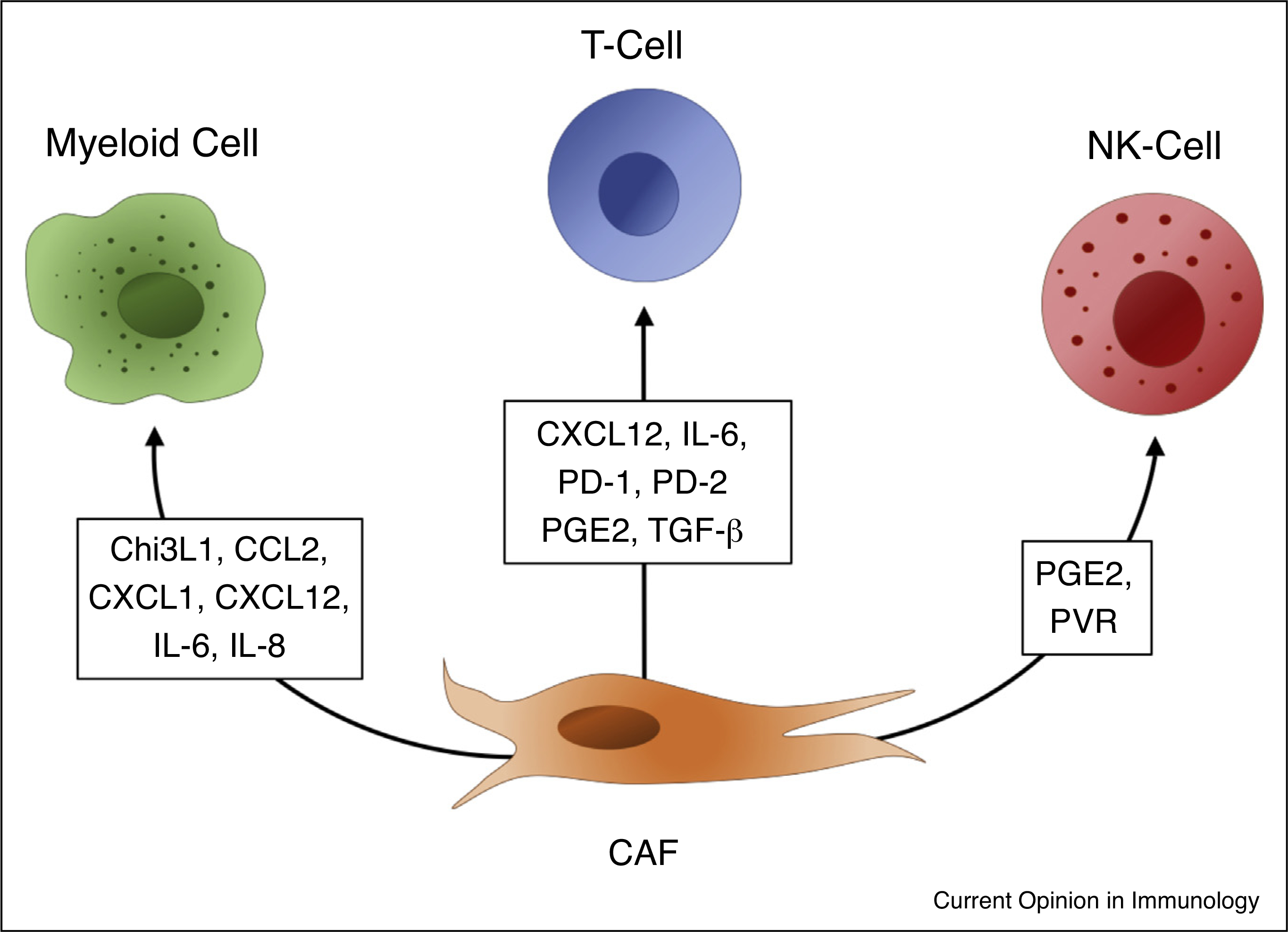
Chitinase 3-like 1 (Chi3L1), chemokine (C-C motif) ligand 2, chemokine (C-X-C motif) ligand 1 (CXCL1), chemokine (C-X-C motif) ligand 12 (CXCL12), Interleukin 6 (IL-6), Interleukin 8 (IL-8), Programmed cell death protein 1 (PD-1), Programmed cell death protein 2 (PD-2), Prostaglandin E2 (PGE2), Transforming growth factor beta (TGFbeta), Polio Virus Receptor (PVR), cancer-associated fibroblast (CAF).

Two of these pathways in particular are well studied in this context: CXCL12/CXCR4 and IL-6/STAT3 (Figure 1). CXCL12 in the TME is largely derived from CAFs and plays an important role in recruiting myeloid cells and promoting an immunosuppressive phenotype. Inhibiting either CXCL12 or its receptor CXCR4, has been shown to decrease intra-tumoral MDSCs [18,36–40]. Further, PGE2 and TGF-β regulate CXCL12/CXCR4 expression and have been proposed as another potential target [38,41,42]. Likewise, myeloid STAT3 is activated in response to CAF-derived IL-6 and is an important regulator of myeloid state that can drive differentiation to regulatory dendritic cells (DCs) [43]. Blocking either STAT3 or IL-6 can disrupt this signaling and reprogram the immunosuppressive milieu of the TME [40,44,45].

Many other CAF-secreted factors are also directly linked to myeloid modulation. CCL2 is produced by a FAP^+^ subset of CAFs and has been shown to attract and to activate STAT3 in myeloid cells [46]. Chitinase 3-like 1 (Chi3L1) secreted by CAFs drives M2 polarization in macrophages [47], and CXCL1 has been implicated as a mediator of CAF-dependent accumulation of MDSCs [48] (Figure 1). It is important to note however, that the contributions of these pathways are context dependent. For example, IL-8/CXCR2 can mediate recruitment of myeloid cells independent of CXCL12/CXCR4 [49,50^••^]. As is often the case, one could theorize these discrepancies arise from varying abundance of heterogeneous CAFs across different models.

### CAF influence on T-Cells

CAF markers correlate with an immuno-tolerant T-cell landscape in the TME, as defined by an increased ratio of FoxP3^+^ to CD8^+^ T-cells, which is also associated with poor clinical outcome [51]. While CAFs can influence adaptive immune cells indirectly through their effect on myeloid cells, CAFs can also exert direct effects on regulatory and cytotoxic T-cells [34].

Subcutaneous tumors developed in mice co-injected with fibroblasts showed a greater ratio of FoxP3^+^/ CD8^+^ T-cells than those in mice injected with tumor cells alone. This effect was attenuated by treatment with anti-IL-6 antibodies consistent with a potential role for CAF-derived IL-6 [51]. Further, fibroblast IL-6 has been shown to drive differentiation of interleukin-17-producing T helper (T_H_17) cells [52] which can be pro-tumorigenic or anti-tumorigenic depending on the tumor type and CAF-mediated signals [53].

Activated fibroblasts can suppress cytotoxic T-cell responses through PD-1 and PD-2 signaling by either expressing PD-L1/2 themselves [54,55], or driving expression of PD-L1/2 on tumor cells via CXCL5 (Figure 1) [56^•^]. TGF-β-associated ECM genes in fibroblasts was reported to be a strong predictor of immunotherapeutic failure and TGF-β blocking antibodies co-administered with anti-PD-L1 therapy significantly improved response to therapy in mouse models [57^••^,^58^]. As with myeloid cells, inhibition of CXCL12 also relieved immunosuppression of T-cells and promoted infiltration of cytotoxic T-cells during anti-PD-1 therapy [37,59].

CAFs also have the capacity for MHC Class I restricted antigen presentation to T-cells. However, rather than activating T-cells, engagement of PD-L2 and FASL on the CAF cell surface can result in killing of antigen-specific cytotoxic CD8 T-cells [60^•^]. A population of MHC-II expressing fibroblasts have recently been highlighted that can present antigen to naïve CD4+ T cells. However, CAF secretion of PGE2 (Figure 1) and a lack of expression of costimulatory molecules by these CAFs appeared to preferentially promote expansion of CD4^+^, CD25^high^, Foxp3^+^ regulatory T-cells (Tregs) [26^••^,^61^].

It is important to note that not all fibroblast-mediated influences on T-cells are immunosuppressive. A limited number of studies provide evidence for T cell-stimulating signaling from fibroblasts. For instance, in some settings IL-6 produced by fibroblasts in response to T-cell exposure enhanced T-cell stimulation [62]. These disparities are likely explained by the heterogeneous nature of CAFs *in vivo* with certain populations performing opposing functions [63]. It also important to consider that these immune-stimulating populations may be misrepresented in past literature as common *in vitro* methods of cultivating fibroblasts can quickly drive a more immunosuppressive state, masking certain populations [62,64].

### CAF influence on natural killer cells

CAFs are able to influence NK cells through both contact-dependent and independent mechanisms. CAFs can block upregulation of NKp44, NKp30, and DNAM-1 triggering receptors as well as the acquisition of cytolytic granules in NK cells stimulated by IL-2, both are important steps in NK cell cytotoxicity [65]. CAF PGE2 was identified as one of the main signaling molecules driving NK cell dysfunction across a variety of cancer types (Figure 1) and inhibitors of either PGE2 or IDO ablated this effect in culture [65–68]. However, as with many other CAF effects, heterogeneity in these responses is observed and studies using CAFs from endometrial cancer promoted NK dysfunction not through PGE2, but through contact dependent mechanisms involving downregulation of cell-surface poliovirus receptor (PVR/CD155) (Figure 1), an important NK cell ligand, on CAFs [69]. Better understanding of the mechanisms through which CAFs manipulate NK cell activation will be imperative moving forward as NK cells become an attractive new target for off-the-shelf adoptive cell immunotherapy [70].

## Strategies to target CAFs to enhance therapeutic efficacy

Targeting CAFs within the TME is a fairly new concept, but given that ECM dysregulation is one of the strongest predictors of failure in immunotherapies such as PD-L1 blockade [58], it has gained considerable interest as of late. Several approaches are being taken to target fibroblasts: 1) taking advantage of the heterogeneity of the population in order to shift the preponderance of pro-tumorigenic populations, including immunosuppressive populations, versus anti-tumorigenic subpopulations 2) targeting pathways that drive differentiation and reprogramming of CAFs, and 3) targeting pathways by which activated fibroblasts negatively influence the TME. (Table 1)

### Targeting CAF heterogeneity

While early attempts to therapeutically target CAFs within the TME failed [19,20], more recent attempts based off our improved understanding of fibroblast heterogeneity have proved more successful. One of the most successful approaches has been in targeting FAP^+^ fibroblasts. The cancer supporting role of FAP-expressing fibroblasts has been known for some time [71]. FAP^+^ cells can both promote tumor progression and present a barrier to immunotherapies through both their production of ECM and direct signaling pathways [72,73]. Multiple different approaches to depleting this population have shown therapeutic promise in preclinical models, with early verification coming from genetic depletion [37,74] and progressing to more translatable approaches like vaccines [75], drug delivering nanoparticles activated by FAP cleavage [76], and CAR-T cells directed at FAP^+^ cells [77,78]. Such treatments in isolation have shown efficacy against cancer and also enhance the activity of conventional chemotherapies and immunotherapies [79]. So far, FAP^+^ populations have been the primary focus in stromal depletion therapies but as more populations become better defined, we will likely see other targets exploited.

### Targeting CAF-specific pathways

Targeted therapies have also been developed and can be classified into two main groups: those that target drivers and those that target effectors. Drugs targeting CAF development and maintenance are aimed upstream and influence factors that drive the phenotypic switch. These include FAK inhibitors [80], Hedgehog inhibitors [81], fibroblast growth factor receptor (FGFR) inhibitors [82], connective tissue growth factor (CTFG) antagonists [83], and TGF-β inhibitors [84], all of which target tumor cells’ ability to activate neighboring fibroblasts. (Table 1)

Effector therapies target pathways already active in CAFs in order to limit their tumor protective abilities. These include vitamin D ligands [85], which reprogram CAFs to a more quiescent-like state, and angiotensin inhibitors [86], which influence CAF matrix deposition, decompressing the tumor, improving its vasculature, and thus making it more susceptible to chemo and immunotherapy. Again in FAP^+^ populations, disrupting pathways that mediate immunosuppression have been successful in tumor models, for example disruption of CXCL12 signaling [37].

Overall, our understanding of how fibroblasts orchestrate and behave across various TMEs is just beginning. Preclinical and clinical studies are showing that fibroblasts are feasible targets for improving immunotherapy response as well as many other therapies. The success of this field will depend upon the discoveries of this coming decade as key gaps are filled so that more targeted therapeutic approaches can be delivered.

## Figures and Tables

**Table 1:** Description of current therapy development underway for stromal targeting in cancer

Target	Name	Drug/biological	Mechanism	Current status

FAK [80]	Defactinib (VS-6063,PF-04554878)	Small molecule	Downstream of integrin signalling	Clinical trials ongoing
Angiotensin receptor [87]	Losartan	Small molecule	Reduces fibroblast contractility	Clinical trials ongoing
Hedgehog [81]	IPI-926 (Saridegib) and Vismodegib	Small molecule	Prevents activation of CAFs	Clinical trials ongoing, some reports lack efficacy
ROCK [88]	AT13148	Small molecule	Contractility	Phase I Clinical trial completed
LOXL2 [89]	Simtuzumab (GS 6624)	Blocking Ab	Anti-crosslinking	Pre-clinical
CTGF [83]	FG-3019	Blocking Ab	Blocks receptor binding	Early phase clinical trials ongoing
Vitamin D receptor [90]	Paricalcitol	Small molecule agonist	‘Normalizes’ stellate cells	Clinical trial started
TGF-β [84]	Multiple	Blocking Ab and small molecule receptor inhibitors	Prevents activation of CAFs and immune-suppression	Phase I, II, and III trials underway
FAP [91]	Multiple antibodies and RO6874281	Blocking Ab or antibody-IL2 fusion	Block FAP+ CAF function, promoting T-cell function	Phase I and II trials underway
FGFR [92]	JNJ-42756493	Small molecule	Prevent activation of CAFs	Phase I and II trials underway
Hyaluronan [93]	PEGPH20	PEGylated recombinant human hyaluronidase	Degrades hyaluronan	Phase III: Enhanced chemotherapy response, did not prolong survival
